# Dental Hints Department

**Published:** 1907-01

**Authors:** B. H. Teague

**Affiliations:** Aiken, S. C.


### ...DENTAL HINTS DEPARTMENT...

CONDUCTED BY B. H. TEAGUE, D. D. S., AIKEN, S. C., TO WHOM ALL CONTRIBUTIONS TO THIS DEPARTMENT SHOULD BE SENT

Operative and Prosthetic Hint.—To make a piece of gold plate stay on the Mellotte's metal die while swaging the top to a crown, wet it.—*Digest.*

It has been found that among other uses the x-ray will restore color to gray hair. Now if it will collect bills.—*Clin. Med.*

Acetanilid and antipyrin act locally on the muscular coat of small vessels as powerfully as ergot.—Hill, *Clinical Medicine.*

It seems the saying, "a man is as old as his arteries," should be altered to "as old as his bowels."—*Clinical Review.*

Morphine is almost entirely excreted by the intestines, only traces by the kidneys, which makes it safe in uremia.—Hill, *Clinical Medicine.*

If all the people in the United States would at once begin chewing properly, three-fourths of the doctors would be put out of business.—Kellogg, *Clinical Medicine.*

Notwithstanding the millions of experiments that have actually been made in the mouth, we do not yet know what is the best local application for obtunding sensitive dentine.—O. W. D., *Milk Review.*

Ribbert's phosphomolybdic-acid-hematoxylin stain after alcoholic fixation which shows the finest fibers of connective tissue. (Whew!)—Dr. V. A. Latham, *Digest.*

Read in *Clinical Medicine* for January about General Anesthesia by Hypodermic Injections. The medicament is "Hyosine, Morphine and Cactine Compound. Abbott."

Cementine Arsenic.—Mix the cement rather thin and place a small drop on a small bit of paper and carry the paper to the cavity with the pliers. Press the place with a burnisher. The paper facilitates adjustment to place and prevents cement adhering to instrument.—Dr. C. B. Warner, *Tri-State Quarterly.*

The bile acts in four different ways: First, it stimulates peristalsis of the small intestines; second, it regulates the degree of fermentation; third, it assists in emulsification and saponification of fat globules in the intestines, and fourth, it destroys putrefactive bacteria.—Dr. E. Talcot, *Items.*

Similarity in Tooth Tissue of Children and Adults.—As I stated years ago, the conditions calling for differences in the treatment of children's teeth and the management of cases in comparison with adults' teeth are due to childhood purely. So far as the tissues of the teeth are concerned we may make fillings in children's teeth just the same as we make them in those of adults, as the tissues of the teeth of the former are hard enough and strong enough.—G. V. Black, *Dental Review.*

With age the bowels, kidneys, lose tone, and as a resullt waste products or toxins circulate in the blood. Between twenty-five and forty, odors eminate from the lungs in the breath, from the armpits and about the thighs. The skin and the lungs are trying to do the work of the bowels and kidneys.—Dr. E. S. Talbot, *Items.*

The first symptoms of pathologic effects due to autointoxication are observable in the alveolar process. In the treatment of interstitial gingivitis, the stomatologist should be mindful of the fact that the patient's system is tending toward disease. —Dr. E. S. Talbot, *Items.*

Nickel-plated Parts.—One of the best methods known for keeping bright the nickel work about the office is to wet a rag with a solution of hypo-sulphite of soda, and wipe the article with it, drying with a soft towel, and then rubbing it with a piece of chamois.—*Bulletin.*

There should be a dentist on every hospital staff, on the medical staff of every home for the orphan or the aged and of every other public or eleemosynary institution. I will go further and state that every institution that requires a physician should also have a dentist.—Dr. F. W. Wiseman, *Era.*

That Terrible Odor.—In opening a tooth with putrescent pulp, where the odor is something terrible, just apply to the cavity a small pledget of cotton previously dipped in terebene and the offensive odor will change to that of attar of roses. Terebene is an antiseptic liquid manufactured by P., D. & Co. An original bottle is sufficient for two or more dentists.—H. B. Davis, *Dental Record.*

Argyrol for Pus Pockets.—After the removal of deposits syringe the pockets with warm water and inject freely a 20 per cent solution of argyrol. This is a thorough non-irritating disinfectant and invariably prevents soreness following the surgical treatment. It is the only drug I find necessary in the treatment of pyorrhea, aside from a good mouth wash.—A. F. James, *Dental Review.*

There is a stage in both $N_2O$ and somnoforme anæsthesia in many patients, after you cease extracting, in which the muscles contract, the fingers are drawn together or forced backward, stiff and rigid; the throat becomes hard and tense, just before consciousness returns, in which the patient can neither spit nor swallow—right at this point, when much hemorrhage is present, is the condition I most dread in my anæsthetic work.—Dr. Geo. Cook, *American Dental Journal.*

Decomposed Pulps.—In case of unexposed pulp in an advanced state of decomposition the application of a paste of paraform (Schering & Co.) and tricresol, in the neighborhood of the pulp, without directly exposing it, results in a slow development of formaldehyde gas which will penetrate the septum of dentin and disinfect the canal and its contents with better results than if attempt is made to open the pulp canal.—E. C. Kirk, *Dental Cosmos.*

Histology of the Pulp.—The histology and pathology of the pulp are simple, in some ways, yet at the same time very difficult. The structure of the pulp is not analogous to any other permanent tissue of the body. It has been likened to the umbilical cord or Wharton's jelly.—Dr. V. A. Latham, *Digest.*

Cementing on an Open Face Crown.—In cementing on an open face crown, paste a piece of court plaster over the labial surface so as to cover the opening and flow wax over this, which will strengthen it so that it will hold the cement in place while placing the crown in position. In this way an open face crown may be attached to a tooth as easily as a full gold crown.—Dr. W. A. Meyer, *Gazette.*

Administration of Gas.—Gas does not act well where liquor and stimulants have been taken. Some people have the mistaken idea that they must "brace up" to have a tooth out. When they come to me with too much of that, I tell them to go away and sober up before they can have gas at my hands. Tobacco and liquor contra-indicate the use of gas.—Dr. Straight, *Dental Register.*

One great source of constipation is lack of water in the system. The feces become dry because the water in the intestinal canal is absorbed. This may be demonstrated by measuring the quantity of urine passed every twenty-four hours. The normal quantity should be three pints or forty ounces. That much water should be drunk each day, including tea, coffee and milk.—Dr. E. D. Talcot, *Items.*

Members of Dental Societies Neglected.—When I look over the field of the dental profession I can only wonder why there are so many dentists, and why such a small number are members of the dental societies. This question can only be answered by placing the blame upon those who are members of the societies in not assuming it as an individual duty to increase the membership in his section of city, county or state.—J. K. Conroy, D. D. S., *Dental Brief.*

First Permanent Molars.—There are eight teeth in the mouth I guard with jealous care: the first permanent molars and the cuspids. When the pulps of the first permanent molars are exposed and the child is under 12 years, I arrive at the question whether to treat them as temporary teeth and extract. I frequently remove one, but never yet consented to extract the other three.—*Dental Review.*

To Remove a Post Crown.—I find that post crowns may be most easily and satisfactorily removed by the use of an excising forcep. Place the cutting edge of the beaks in the joint between the root and crown—one on the lingual and one on the buccal side, as in the excision of a tooth. Now, by applying a little force and twisting the forceps a little, the sharp beaks force themselves into the joint and because of their wedge shape the crown gradually ''lets go'' until it is loose.

Post-crown bridges may be removed in the same manner by loosening each abutment a little at a time.—J. F. Nelson, *Western Dental Journal.*

Amalgam Fillings.—The best method of making amalgam fillings tight is to use all the force we can and pack it along the walls with a very small instrument. The amalgam must be hard enough. It is not so difficult to make walls that are practically perfect. It is easy to make walls that will not show spaces with the microscope. Still we can make fillings that will do our patients much good, often where a gold filling would not answer as well.—G. V. Black, Chicago, *Dental Review*

Malodorous Rubber Dentures.—To obliterate the spaces which are always found under section teeth when they are mounted on rubber, and in which mucus and food debris collect and putrefy; to secure a better anchorage for the pins in the rubber; to enhance the durability of the denture and its cleanliness, doing away with the interstices which receive and retain putrefying organic matter, vulcanize by Snow's new process, using the Whitney flask with extra long bolts and springs.—*Dental Brief.*

Pearline makes an ideal separating material, dissolved and colored with red ink or a bit of indellible pencil. A dash of the powder into the boiling water for removing the wax from an investment acts like magic; or for cleansing the hands first lather them with a good toilet soap and on to that apply some of the powder; work it up thoroughly, getting it into all crevices, then apply the brush or sponge with water and the hands are clean before you know it; dry them and apply not more than four or five drops of 2 parts glycerine and 1 part Pond's Extract perfumed and your hands are ready to work for anybody.—Dr. G. A. Dille, *Items.*

Silver Nitrate with Cement Fillings.—A cement filling, zinc oxyphosphate, placed upon a surface treated with nitrate; will, for some reason, last a great deal longer and will be a great deal better mass than the same mass not having the peculiar effect it gets from this film of silver albumite. A great many, no doubt, have seen the effect of silver nitrate upon a surface which has been infected to a very slight depth. That in time will become a polished black surface and further decay will never result. Now if in deeper cavities we can make partial preparation and apply silver nitrate, and have it last longer than otherwise, it is worth knowing.—W. V. B. Ames, *Dental Review.*

Root Canal Filling.—Having root ready for filling, and for the most cases having the rubber dam in place—securing a good view and dryness—I use the oxide of zinc or the powder of ox-para and trikesol, adding carbonized cotton to thicken the paste, when using it for the back teeth, thus making the mixture more readily carried to place, not, however, without a good degree of patience and considerable perseverance, the sense of touch and your judgment telling you when to cease the pumping, and after this, if you desire, a guttapercha cone finishes the filling.

In this you have a continuance of the medication that has rendered your tooth root comfortable, and in tender teeth it has proved itself very valuable in my practice.—Grafton Munroe, *Dental Brief.*

Objection to Ether.—The chief objection to ether that is held to be a real danger—that of the large amount needed, which may seriously tax the eliminative organs—may be largely removed by preceding its use by either ethyl chlorid or nitrous oxid. The fact that it is not a heart poison gives it a place of permanence as the safest known agent for a profound, continued effect. It induces practically no disturbance of circulatory conditions.—*Register.*

A solution of equal parts of chloroform and crystallized chloride of zinc, as suggested to me by Dr. Hofheinz, with an addition of cocaine and libitum, has given me the best results in cavities of decay. For superficial sensitiveness at the necks of the teeth, nitrate of silver, applied in substance or concentrated solutions and the part protected afterward for a few hours by a covering of Fletcher's chloride of zinc applied in the same way gives especially good results without the discoloration.—Dr. W. D. Miller, *Review.*

Hydrogen Peroxide in Skin Cosmetics.—H. Keuhl states that glycerine and rose water used in the treatment of chapped hands is improved by the addition of hydrogen peroxide, thus: Glycerin, 2; rose water, 2; hydrogen peroxide, 1. Mix. It may also be mixed with ointments for the treatment of skin affections. Intimately mixed with lanum, it produces a smooth, soft, white cream, which may be used to bleach the skin. A mixture of equal parts of lanum and zinc ointment, combined with hydrogen peroxide also forms a useful ointment for the same purpose.—*Stomatologist.*

We take gold in any form or quantity, from one to twenty-four carat, and melt it with silver, alloying it down to ten carat. It is then granulated in small shot by pouring into water when in a melted state. The granulated gold is then boiled in a retort on a sand bath in nitric acid until the silver and base metals are dissolved, the gold remaining in the shape of brown mud, which is then washed, dried and melted. The result is what we call parting gold, which is .996 or .998 fine, and it is from this that most gold foils are manufactured.—John Hood, *Gazette.*

Reface Gum-Sections.—The ground faces of the surfaces that makes the joints of a set of some makes of gum teeth are porous, and because of this condition, whether worn for a week or a year, drink in saliva with matter held in solution and suspension, with the result that if the teeth are reset without further treatment this organic material is carbonized during vulcanization, giving the joints of the remounted set an appearance as offensive as if rubber-filled. Chemical treatment of the teeth when taken from the old plate helps matters somewhat; but the way of ways is to reface the ground surfaces by means of a fine wheel—corundum, carbonundum or even a fine sand paper disk. This treatment removes all of the affected and infected material, and yet not enough of the porcelain to make any appreciable difference in the spaces between the sections.—*Dental Office and Laboratory.*

Operative Procedure for Excavating Sensitive Cavities.—There is a distinct difference in the sensitiveness, dependent on the manner in which the dentine is cut. Success in many cases may be achieved by cutting the dentine in the following manner: Instead of using the usual bur and cutting transversely, cut vertically to the axis of the dentine, and with a drill with a flat point cut a series of small holes, and the operation can then be continued with half the pain that would be otherwise occasioned. What generally troubles most people is to get an anchorage, but by drilling a series of small holes the dentine may be cut with very little pain compared to that caused by using a drill in the ordinary manner.—H. L. Schaffner, *The Dental Surgeon.*

Making a Matrix.—Our matrix is prepared by forcing the center of a large piece of 1-1000 platinum foil, annealed, into bottom of cavity with ball of cotton. This removed, the matrix is held in place with a strip of rubber dam and burnished to accurate fit. The final burnishing direct on platinum foil at margins makes it thinnest here, giving the best possible joint. Holding the matrix firmly with fingers over margin, the excess foil is bent free. Then the matrix is easily teased out.—Dr. C. E. Abbott, *Items.*

No Pyorrhea with Normal Occlusion.—I do not remember that I have ever seen a case of pyorrhea in the mouth with normal occlusion, and I think that there is almost no irritation of the gum tissue because nature has given us an apparatus which is really self-cleansing, as Dr. Black puts it, the natural run of the food will keep the surface clean. When a few teeth are lost the other teeth soon drift into bad occlusion, and every protected surface will serve to accumulate ptomanies, and where these accumulate there will nearly always be inflammation.

Dorrance alloy consists of pure silver one part, pure zinc two parts, pure copper three parts.

In making the alloy melt the silver and copper together and when in molten state push the zinc into it quickly, a small quantity at a time, and between times cover with borax.

The following is the formula for 18k. solder:

| | |
|---|---|
| Coin gold - - - - - - - | 30 grs. |
| Dorrance alloy - - - - - - | 4 grs. |
| Pure silver - - - - - - | 3 grs. |
| Pure copper - - - - - - | 3 grs. |

The following is the formula for 14k. solder:

| | |
|---|---|
| Coin gold - - - - - - - | 30 grs. |
| Dorrance alloy - - - - - - | 10 grs. |
| Pure silver - - - - - - | 3 grs. |
| Pure copper - - - - - - | 3 grs. |

—*Review.*

Bubbles in Porcelain.—I believe the reason I do not have bubbles is that in mixing the body I spatulate it with a great deal of force, mix and incorporate it thoroughly, grinding it down with a strong spatula, and when it comes to settling the body, settling it vigorously in one direction. So many, when they have a crown to inlay will settle the body in one direction, then they will turn it over and unsettle it again, and rasp it up, change the original settling of that body until—why shouldn't there be bubbles?—Dr. J. D. Patterson, *Western Dental Journal.*

Adhesion of Cement.—One can easily be deceived as to the adhesiveness of a cement, from noticing one specimen firmly adhering to a glass slab some hours after the mix, and another being loose or easily removed. If carefully investigated it will be found that adhesion in the first case comes from imperfect crystallization, there being gummy free acid next the glass, while in the second case the crystallization has been such that no gummy acid is present. Touching the tongue to the under surface of the pellet will give the evidence. Adhesion to glass after a few hours is a bad rather than good indication.—*Summary.*

They were both *purified, beaten* and *annealed* in the same manner, but just previous to marketing a part of the lot is passed through ammonia fumes and marked non-cohesive simply because these fumes form an imperceptible film which, while not affecting the purity of the gold itself, prevents intimate cohesion of the different layers of gold. From this we may see that a pellet of cohesive gold may be made non-cohesive by exposing it to the influence of ammonia gas, and this pellet thus rendered non-cohesive may in turn be made cohesive by driving off the gas with heat. This latter process is what we actually accomplish in our every day operations with non-cohesive foil when we heat it and it is in no way intended to anneal the gold.—Dr. M. L. Ward, *American Dental Journal.*

I have been asked at different times and by some prominent members in the profession to write something that would be a decided "knock" on the various "nostrums" that are on the market under such fancy names as "Arnoda," "Ox-Para," "Pustoline," "Ab-Konker," "Nerve Quietus." etc. Some of these preparations, I presume, have been compounded in good faith, and while I do not wish to put myself on record as a "knocker," I will say that all things being considered, to use remedies, whose formulæ we do not know, is decidedly unscientific and unprofessional.

All of the above mentioned preparations have a similar composition. The liquid, as a rule, consists of equal parts of formalin and creosote and the powder of dry alum, thymol and oxide of zinc.—Dr. E. T. Leffler, *Magazine.*

Cold Soldering.—Dr. Beebe says that fresh amalgam can be made to stick to an old amalgam filling by merely coating the freshly-exposed surface of the latter with chlorhydric acid.

Dr. Sibley added that the same object could be attained (according to the late Dr. Palmer, he thought) by treating the freshly-exposed surface with silver nitrate.—*Dental Office and Laboratory.*

Preparing Platinum for the Matrix.—In using platinum as a material for matrices, it is well to have it of at least two thicknesses—one for commercial, which is supposed to be 1-2000 of an inch in thickness. This can be annealed and oiled and folded together, and with a good set of rolls can be reduced to probably one-half its thickness, and is much better for small inlays than the thicker platinum. Various methods for annealing have been suggested. One very good way is, in the furnace, heating it up to two thousand degrees or more, then allowing it to cool gradually. Another very excellent way is the oxyhydrogen blowpipe, which heats it very rapidly and very hot, and is more convenient on account of saving time, and the result is equally as good.

The platinum which has been doubled and passed through the rolls for the purpose of reducing the gauge, is glazed on the surface which comes in contact with the steel roller, while the other side has a frosty or velvety surface. The frosted surface should be placed in contact with the tooth, the glazed side in contact with the porcelain, as it strips from the inlay more easily than the frosted side.—*Western Dental Journal.*

Campho-Phenique as a Local Anesthetic.—All operations in the mouth should be preceded by the application of a local anesthetic. In many mouths nausea, even vomiting, is caused by the insertion of fingers, instruments, or impression-trays.

To avoid this especially is campho-phenique indicated to obtund local sensation and prevent pain incident to the removal of calculus, polishing teeth, wedging, applying and ligating the rubber dam, removing loose roots, and to prevent nausea, gagging, etc., prior to taking impressions.

Nothing to the writer's knowledge acts so nicely as ten grains of finely powdered cocain added to one fluidounce of camphophenique (original package). This makes approximately a two per cent solution, which, when applied on a large-sized pledget of cotton to the entire mucous surface of the mouth, completely obtunds local sensation and overcomes nausea.

In cases of odontalgia from an exposed pulp a warmed application of this solution on a pellet of cotton usually relieves the pain, and for treating sensitive cavities preparatory to filling it is of equal value when the remedy is sealed in the cavity with temporary stopping of gutta-percha.—Dr. B. L. Thorpe, *Brief*.

I showed that interstitial gingivitis is a disease and that pyorrhea alveolaris is the result of a disease and not a disease itself. So bear in mind, please, this point, that interstitial gingivitis is the disease that you must treat. You all know very well that you can not have pus infection in any part of the body without an inflammation. That is fixed. Why monkey with pyorrhea alveolaris? Drop your pyorrhea alveolaris and treat your disease and you will not have any pyorrhea; treat your interstitial gingivitis and let your pus alone, let your pockets alone, except to cleanse the teeth locally. Remove all the irritation about them, and treat your inflammation and the pus will take care of itself. The profession commences at the wrong end. They treat the pus condition and when they stop that, do not know anything about the interstitial gingivitis which is still there.—Dr. E. S. Talbot, *Items*.

